# Were deaths recorded in Brazil due to cystic fibrosis or pulmonary fibrosis? A data-based analysis

**DOI:** 10.3389/fmed.2024.1459785

**Published:** 2024-08-21

**Authors:** Leonardo Souza Marques, Mônica Cássia Firmida, Fernando Augusto Lima Marson

**Affiliations:** ^1^Laboratory of Molecular Biology and Genetics, University of São Francisco, Bragança Paulista, São Paulo, Brazil; ^2^Laboratory of Clinical and Molecular Microbiology, University of São Francisco, Bragança Paulista, São Paulo, Brazil; ^3^LunGuardian Research Group—Epidemiology of Respiratory and Infectious Diseases, University of São Francisco, Bragança Paulista, São Paulo, Brazil; ^4^Department of Integrated Medical Sciences, University of the State of Rio de Janeiro, Cabo Frio, Rio de Janeiro, Brazil

**Keywords:** cystic fibrosis, epidemiology, interstitial pulmonary diseases, idiopathic pulmonary fibrosis, medical semantics

## Introduction

Cystic fibrosis [CF, OMIM (Online Mendelian Inheritance in Man) n° 219700] is a genetic condition associated with the presence of pathogenic variants in the *CFTR* (*Cystic Fibrosis Transmembrane Regulator*) gene (1–3). Historically, the clinical phenotype of the illness was associated with the presence of salty sweat, which resulted in the use of chloride ion quantification in the sweat as a diagnosis criterion (4). In Brazil, although its higher prevalence is ascribed to individuals of Caucasian descent, it has also been observed in other ethnic and racial groups (5). In the last few years, due to advancements in its treatment, the survival rate of patients with CF has increased (6, 7). Therefore, individuals who would probably die at birth or in the first few years of their life were seen to live up to adolescence, and more recently, to adulthood (8). However, there are still discrepancies in the life span among different populations of patients with CF and among those in the same population. For this reason, greater importance has been given to the patients' genetic profile as well as their access to healthcare, diagnosis, and treatments using precision and personalized medicine (9–11). Taking these factors into consideration, we sought to evaluate the death rate of patients with CF and determine the most affected age groups in Brazil. Please see the graphical abstract in the Supplementary material Presentation 1.

**Figure 1:**
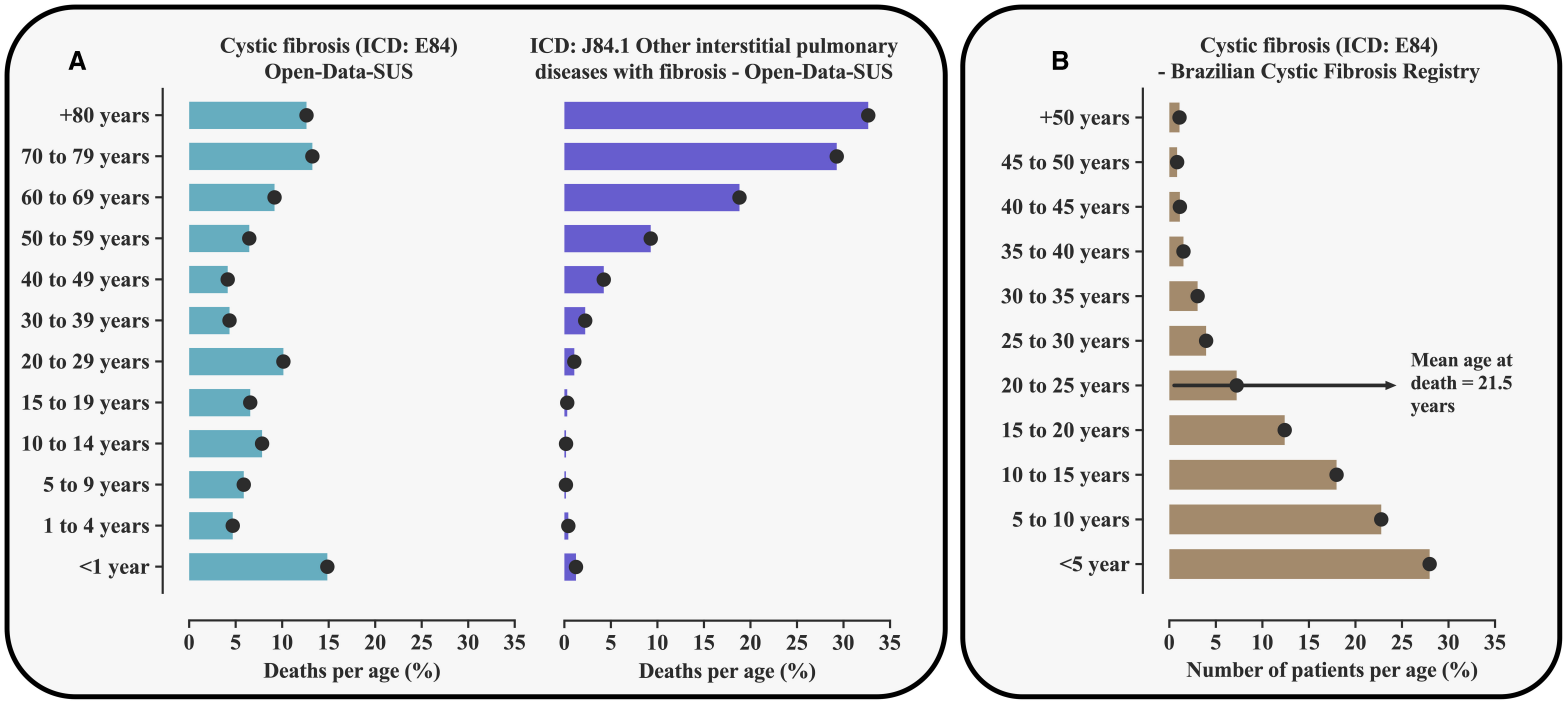
Percentage of the number of deaths resulting from cystic fibrosis [International Classification of Diseases (ICD): E84] and ICD: J84.1 associated with interstitial pulmonary diseases including mainly idiopathic pulmonary fibrosis according to the Death Information System (SIM, from Portuguese *Sistema de Informações sobre Mortalidade*, Open-Data-SUS) in Brazil and taking into account age groups **(A)**. The figure also shows the distribution of patients with cystic fibrosis assisted in Brazil in 2021 taking into account age groups **(B)**. The data used can be obtained from TabNet (https://datasus.saude.gov.br/informacoes-de-saude-tabnet/) and from the Brazilian Cystic Fibrosis Registry (http://portalgbefc.org.br/), or upon request to the authors. The age cut-off points are presented according to the distribution used in each of the registers included. For the calculation of the percentage of deaths, cases recorded in the Death Information System that did not have the patients' age were disregarded.

To achieve the study aim, we evaluated data stored in the Brazilian death register pertaining to patients with CF [International Classification of Diseases (ICD): E84], which showed 3,837 deaths within a period of 27 years. Curiously, unlike the literature reports (4, 12, 13), most deaths were recorded among adults or old individuals (Supplementary Table 1, Figure 1A). These data were in conflict with the epidemiological data as recorded in the Brazilian Cystic Fibrosis Registry (REBRAFC, from Portuguese *Registro Brasileiro de Fibrose C*í*stica*, 2022) (Supplementary Table 2, Figure 1B). According to the Brazilian Cystic Fibrosis Registry, in 2021, the CF patients' population was predominantly young and 74.4% of the individuals were younger than 18 years. Also, in 2021, 50 deaths resulting from CF were reported in a population whose mean age was 21.5 ± 9.9 years. In contrast, in the Open-Data-SUS record of deaths, its Death Information System registered 272 deaths only that year, out of which 51 individuals who died were older than 80 years [Brazilian Death Information System—SIM (from Portuguese *Sistema de Informações sobre Mortalidade*), Open-Data-SUS]. Considering the Open-Data-SUS as the source of information about the health of the Brazilian population and the evidence of errors found in death registers by this research, in which CF was possibly confused with idiopathic pulmonary fibrosis as the cause of death, we concluded that this is an urgent issue, needing a discussion as it has become an unavoidable ethical commitment.

The Brazilian Cystic Fibrosis Registry, which stores information provided by the Referral Centers, is the main source of information about CF in the Brazilian population. The register efficiently monitors patients with this disease aiming to promote better access to diagnosis and treatment and is also used as a reference for the knowledge of this disease in the population. Considering the excellent maintenance of their register regarding the number of cases and patients' follow-up, the contrasting data observed is alarming. The conflicting data could be due to some factors associated with data tabulation, which include: (a) typing mistakes when entering information in the Open-Data-SUS System and (b) poor data management and incorrect ICD record due to phonetic similarity, as for example ICD: J84.1 associated with interstitial pulmonary diseases including mainly idiopathic pulmonary fibrosis, which mainly affects the elderly (Supplementary Table 3, Figure 1A). Such semantic confusion might result from a lack of technical knowledge about CF, mainly of medical doctors that give assistance to adults, including pneumologists, since not long ago it was quite rare to see adults affected by this disease in the country. The illness was found to essentially occur in the pediatric age range. In addition, pulmonary diseases that develop with fibrosis may, in addition, to give rise to tomographic signals possibly found in fibrosing pulmonary diseases, which might include the presence of fibrosis and cysts (14).

## CF disease: a brief review

CF is one of the most common genetic diseases. It is an autosomal recessive inheritance illness that affects multiple organs and shows a wide spectrum of clinical manifestations, including chronic obstructive pulmonary disease, sinusitis, steatorrhea, malnutrition, sweat electrolyte loss, and obstructive azoospermia (15, 16). It is progressive and shows great severity potential (15, 16). The pulmonary disease is characterized by chronic and productive cough, dyspnea, and “repetition pneumonias” (exacerbations) resulting from chronic infections, which provoke bronchiectasis and other structural alterations that compromise the pulmonary function and might cause complications such as hemoptysis, pneumothorax, and respiratory failure (15, 16). The earlier the patients are diagnosed and treated in referral centers, the better their quality of life and survival rates are. Although currently most people have access to the diagnosis from the positive neonatal tracking, which in Brazil is possible by means of serum immunoreactive trypsinogen dosage in the neonatal heel pricking test (17), some patients depend on the medical doctor's technical ability to suspect CF in this diagnosis. The diagnosis becomes easy when CF is present in the family but gets difficult when it should be made only from the signs and symptoms regarded as suspicious. Except for the meconium ileus, most of the initial clinical manifestations of CF are not pathognomonic and are found in other common childhood diseases such as pneumonia, asthma, and food allergy. For this reason, it can be considered a rare disease (mean incidence of one per 10,000 people in Brazil). Due to the stigma of being a severe and potentially lethal disease, the CF hypothesis is many times disregarded when it should be considered. This causes a series of irreversible damages to those affected by the disease, which increase their morbidity (18). The need for more complex care tends to increase proportionally with the patients' age (4). In developed countries such as those in Europe, ~51.2% of individuals with CF are adults with a survival period of ~50 years (19). However, in developing countries, the lifespan is shorter, for example, ~21.5 years in Brazil (20).

CF remains incurable, but advances in treatment have significantly extended the lifespan and improved the quality of life of many patients (6, 7, 21). Effective management requires a multidisciplinary healthcare team, including CF specialists, to initiate the treatment promptly upon positive newborn screening, even before the confirmation of diagnosis (17). Treatment strategies focus on airway clearance techniques, medications to enhance CFTR protein function, and interventions such as respiratory and nutritional support or surgery when necessary (22, 23). The comprehensive CF care team typically comprises specialists in pulmonary medicine, endocrinology, gastroenterology, fertility, genetics, nursing, nutrition, palliative care, pharmacy, physical therapy, psychology, respiratory therapy, social work, and transplant care (23).

In CF, airway clearance methods, such as oscillatory positive expiratory pressure devices and chest physical therapy, aid in mucus clearance, reducing infections, and improving respiratory function along with the use of bronchodilators and mucolytic agents (22, 23). CF medications target various aspects of the disease, including antibiotics for infection management, anti-inflammatory drugs such as ibuprofen or corticosteroids to reduce inflammation, and CFTR modulators to enhance defective protein function based on genetic pathogenic variants (9, 22, 23). CFTR modulators, administered orally, require genetic testing to determine suitability and may interact with other medications and cause side effects. The approved CFTR modulators include Elexacaftor–Tezacaftor–Ivacaftor (a triple combination medicine approved for adults and children aged two and older), Ivacaftor alone (approved for adults and children as young as four months old), Lumacaftor–Ivacaftor combination (approved for people who are at least one year old), and Tezacaftor–Ivacaftor combination (approved for people as young as four months old, tailored to specific pathogenic variants of the *CFTR* gene) (24–27). Additional therapies such as oxygen therapy, pulmonary rehabilitation, ventilator support, and extracorporeal membrane oxygenation are employed for severe respiratory complications (26). Surgical interventions, including lung or liver transplants, are considered for advanced cases of CF-associated organ dysfunction (26, 28).

## Idiopathic pulmonary fibrosis: a brief review

Pulmonary fibrosis involves a wide group of diseases characterized by progressive healing (fibroblasts) and progressive impairment. Idiopathic pulmonary fibrosis, as the name suggests, is a disease whose etiology has not been fully elucidated. It is the most common among fibrosing pulmonary diseases and has a debilitating character, with high physical and emotional morbidity (29). Suitable diagnosis involves the recognition of clinical data and identification of typical patterns of alteration in high-resolution thorax tomography and, sometimes, in pulmonary biopsy. Morphologically, interstitial remodeling of alveolar spaces is observed, with fibroblast foci, hyperplasic skin cells, and honeycomb-like cysts (30). The main manifestations are cough and dyspnea, which tend to worsen progressively while pulmonary fibrosis develops and the lung function impairment increases (31). The patients' mean age ranges between 65 and 70 years and its incidence increases over time (32). It mainly affects men, and environmental (smoking and metal inhaling) and genetic risk factors are better known to be associated with the development of this illness (33). For methodological reasons, for example variations in definition, the incidence of idiopathic pulmonary fibrosis is not well-defined. The estimates range from 0.09 to 4.51 per 10,000 individuals, and the affected individuals' survival depends on the progression rhythm and the degree of pulmonary impairment (31, 33, 34). It is very important to distinguish idiopathic pulmonary fibrosis from other specific illnesses that might develop with pulmonary fibrosis. Some of these cases may also have a rapidly progressive clinical course, leading to death before the patient can access a specialist doctor. This is another issue that can bias the Open-Data-SUS data regarding the cause of death determined as “idiopathic pulmonary fibrosis” (35). Idiopathic pulmonary fibrosis continues to have poor survival rates (36). First-generation antifibrotics, Pirfenidone and Nintedanib, which received approval more than a decade ago, have shown efficacy in slowing disease progression and prolonging survival in patients with idiopathic pulmonary fibrosis, as well as showing promise in other fibrotic lung disorders, such as, for example, pneumonitis with fibrotic hypersensitivity or progressive pulmonary fibrosis (36–39). Despite these advances, most clinical trials targeting idiopathic pulmonary fibrosis in recent years have failed to achieve their primary endpoints, highlighting an ongoing urgent need to identify new agents or treatment strategies capable of stopping disease progression (36). Currently, several new drugs are in various stages of clinical development, predominantly in phase I and II trials, with only a limited number in phase III trials (36).

## CF disease and idiopathic pulmonary fibrosis: similarities and differences

Although both conditions, CF and idiopathic pulmonary fibrosis, involve pulmonary fibrosis—characterized by excessive extracellular matrix deposition due to dysregulated wound and connective tissue repair response—as a consequence of disease progression, their etiologies and clinical presentations differ markedly (40). Idiopathic pulmonary fibrosis primarily affects older adults, and its precise etiology remains elusive (41, 42). On the contrary, CF is a genetic disorder that typically manifests early in childhood (17). Symptomatically, idiopathic pulmonary fibrosis manifests with progressive dyspnea and a dry cough (41, 42), while CF is characterized by recurrent lung infections and multi-organ involvement, including the digestive system (2, 43). Treatment strategies for idiopathic pulmonary fibrosis focus on the management of symptoms and the attenuation of disease progression (36–39), while CF requires a comprehensive therapeutic approach targeting respiratory, digestive, and other systemic manifestations (9, 22–27).

Although definitive cures for both pulmonary fibrosis and CF remain elusive, ongoing advancements in medical research are continuously enhancing treatment modalities and improving patient quality of life. In CF disease, significant progress has been made in recent years with the development of therapies capable of modulating the expression of the CFTR protein (24–27). Furthermore, the pathogenesis of idiopathic pulmonary fibrosis involves epithelial injury and subsequent infiltration of monocytes, which differentiate into monocyte-derived alveolar macrophages, ultimately leading to fibroblasts resistant to apoptosis (44–46). On the other hand, CF arises from pathogenic variants in the *CFTR* gene, resulting in an absent or dysfunctional CFTR protein (47). This dysfunction disrupts chloride ion transport across epithelial cell membranes, causing excessively salty sweat and thickened secretions (47). The strategic location of the CFTR protein in epithelial cells differs between various tissues of the human body (47). In the lungs, this disruption leads to dehydration of airway surface fluid critical to maintaining ciliary function and mucociliary clearance (47). Consequently, there is a detrimental cycle of mucus retention, recurrent infections, and chronic inflammation (47). Although this pathophysiological concept is widely accepted, controversies persist with respect to multiple aspects of this cascade (47).

## Enhancement of the scientific and medical issue: where it went wrong and how we can resolve this error

Good clinical practices for the diagnosis and care of CF patients and those with idiopathic pulmonary fibrosis require medical abilities and competences that go beyond mere theoretical knowledge about these illnesses. The flaws in the care and recording of these diseases also arise from weaknesses in professional qualification. Lack of clinical abilities or, as pointed out by Herbert L. Fred, hyposkillia is a serious problem in Brazil too, and it raises greater concern with the sharp increase in the number of medical schools in the last few years (48). That author used the term to describe medical doctors who cannot obtain a proper medical history, cannot perform a reliable clinical examination, cannot critically evaluate the information gathered, find it difficult to develop good clinical reasoning and plan the treatment, and also show poor communication skills (48).

Considering that clinical manifestations and the age group mostly affected by CF and idiopathic pulmonary fibrosis are quite different, we suggest that the confusion in relation to the death data is predominantly semantic. However, the consequences of such a mistake for the Brazilian public health are damaging. At least, the wrong data alter the CF lethality indicator, thus increasing the stigma of association between CF and death. They also hamper the use of such official data in health management, care, teaching, and research (5, 49). Similarly, the quality of the care provided to individuals affected by these conditions remains an area of concern, and the lack of technical knowledge might delay a proper diagnosis and treatment, thus compromising the patients' quality of life and survival rates. CF and idiopathic pulmonary fibrosis must be better discussed and clarified.

In an era when genetic (and other) influences have been increasingly identified in adult life illnesses and when children with rare and complex conditions enjoy a longer lifespan, it is necessary to promote a radical paradigm change in medical education, at all levels of qualification. This education must be more holistic and connective, and its object must be individuals who are born and might live up to 100 years old, their life context, and the environment. The common reductionism of the current education focuses on diseases and, even worse, they are fragmented as “children's illnesses” and “adults' illnesses.” As pointed out by the medical doctor and biologist Siddartha Mukherjee in the book *The Song of the Cell*, knowledge in the most varied health areas has become deeper and more specific; however, there are still gaps in relation to its connections and interdependences (50). We know more and more musical scores, the metaphors for deep knowledge about cells, but we still do not work as an orchestra (50).

In this context, the results found in this research suggest the urgent need for technical qualification and continuous education of medical doctors for a correct diagnosis of CF and pulmonary fibrosis, whose confusion might compromise the information generated and cause harm to patients.

## Supplementary material

Presentation 1 Graphical abstract.
