# Supplementary material for: Were deaths recorded in Brazil due to cystic fibrosis or pulmonary fibrosis? A data-based analysis
Source: Front Med (Lausanne). 2024 Aug 21;11:1459785. doi: 10.3389/fmed.2024.1459785 (PMC11382496; doi:10.3389/fmed.2024.1459785)

Were deaths recorded in Brazil due to cystic fibrosis or pulmonary fibrosis? A data-based analysis

Fibrosis: Medical Semantics

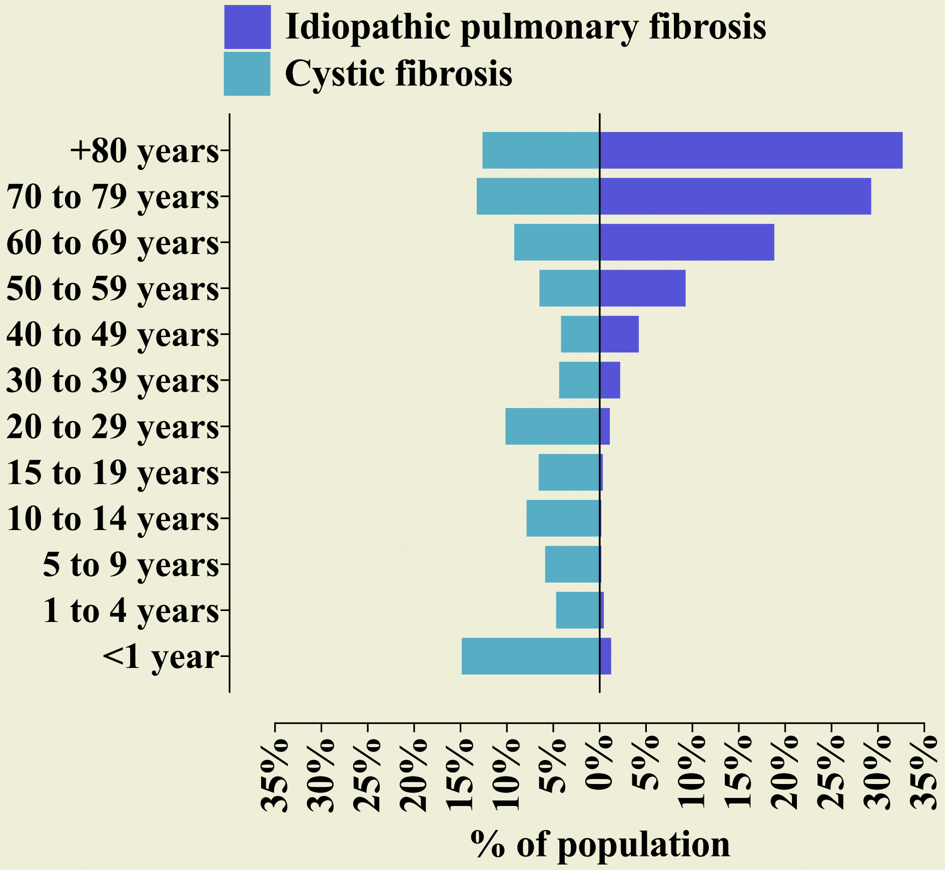

Percentage of the number of deaths resulting from cystic fibrosis [ICD: E84] or interstitial pulmonary diseases including, mainly, idiopathic pulmonary fibrosis [ICD: J84.1] according to the Death Information System in Brazil considering age groups.

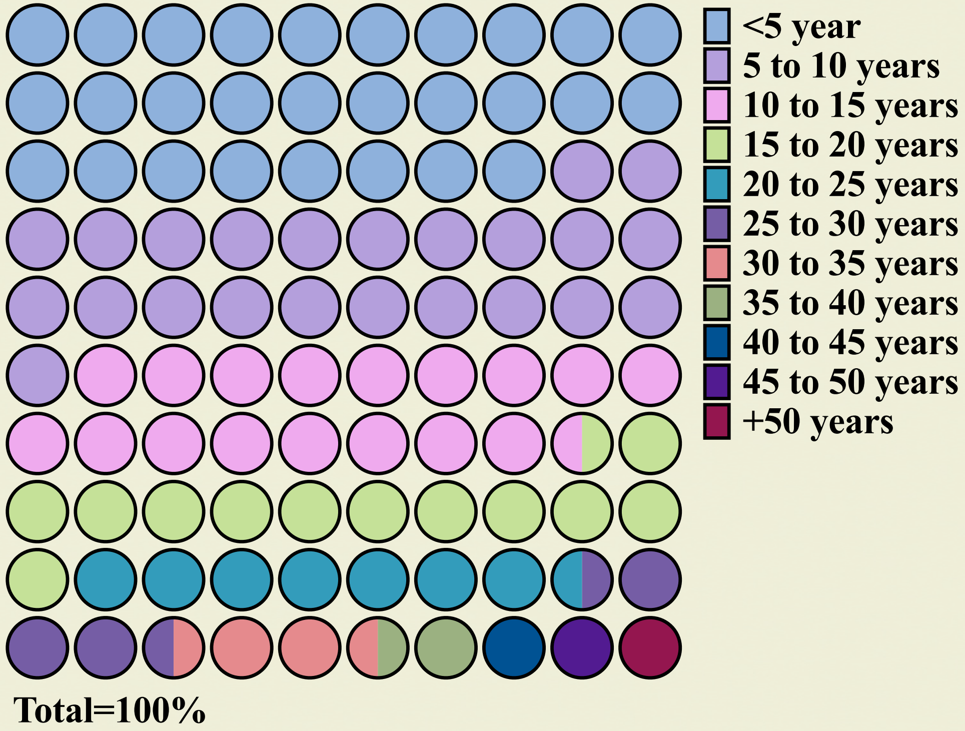

Distribution of patients with cystic fibrosis assisted in Brazil in 2021 considering age groups.

The study underscores the necessity of implementing educational programs aimed at enhancing disease coding accuracy. It emphasizes the critical importance of improving diagnostic procedures, given their profound impact on health policies.

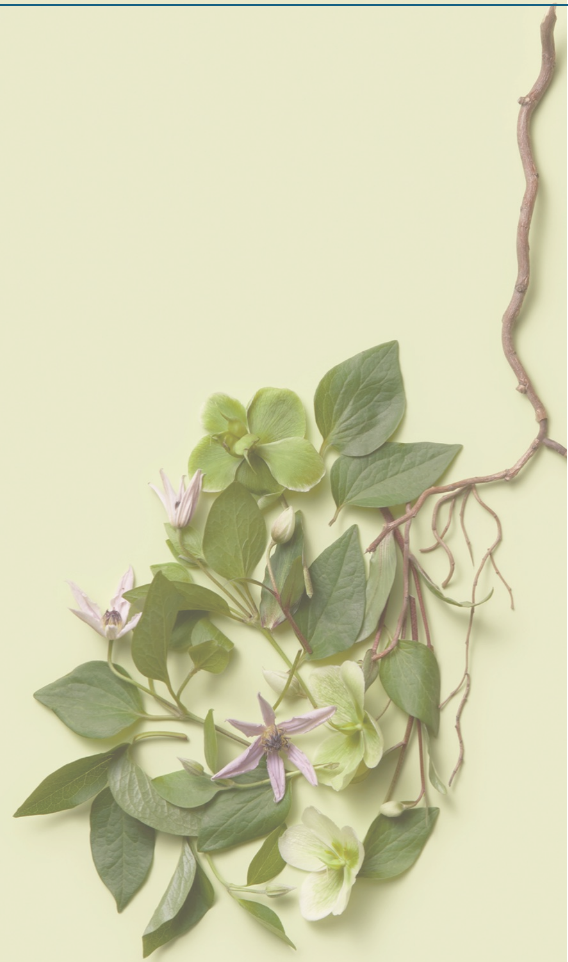

Supplement: Presentation 1 — Graphical abstract. [file Presentation_1.pdf]
