# Supplementary material for: Were deaths recorded in Brazil due to cystic fibrosis or pulmonary fibrosis? A data-based analysis
Source: Front Med (Lausanne). 2024 Aug 21;11:1459785. doi: 10.3389/fmed.2024.1459785 (PMC11382496; doi:10.3389/fmed.2024.1459785)
Supplement: Supplementary file 2 [file Data_Sheet_1.docx]

**Supplementary Material**

**Title:** Were deaths recorded in Brazil due to cystic fibrosis or pulmonary fibrosis? A data-based analysis

**Short title:** Fibrosis: Medical Semantics

Leonardo Souza Marques ^1,2,3^; Mônica Cássia Firmida ^3,4^; Fernando Augusto Lima Marson ^1,2,3,*^

^1^ Laboratory of Molecular Biology and Genetics, University of São Francisco, Bragança Paulista, São Paulo, Brazil

^2^ Laboratory of Clinical and Molecular Microbiology, University of São Francisco, Bragança Paulista, São Paulo, Brazil

^3^ LunGuardian Research Group – Epidemiology of Respiratory and Infectious Diseases, University of São Francisco, Bragança Paulista, São Paulo, Brazil

^4^ Department of Integrated Medical Sciences, University of the State of Rio de Janeiro, Cabo Frio, Rio de Janeiro, Brazil

* Corresponding Author: [FALM] Fernando Augusto Lima Marson, BSc, MSc, Ph.D.

University of São Francisco; Health Sciences Postgraduate Program; Laboratory of Molecular Biology and Genetics, Laboratory of Clinical and Molecular Microbiology, and LunGuardian Research Group – Epidemiology of Respiratory and Infectious Diseases.

Avenida São Francisco de Assis, 218. Jardim São José, Bragança Paulista 12916-900, São Paulo, Brasil. Phone +55-19-999752911.

E-mail: fernandolimamarson@hotmail.com and fernando.marson@usf.edu.br.

**E-mails and ORCIDs:**

**LSM:** leonardo.marques@mail.usf.edu.br

ORCID: 0000-0002-3379-2388

**MCF:** mfirmida@gmail.com

ORCID: 0000-0003-1741-2952

**FALM**: fernandolimamarson@hotmail.com and fernando.marson@usf.edu.br

ORCID: 0000-0003-4955-4234

**Statements**

**Funding:** The author(s) declare that no financial support was received for the research, authorship, and/or publication of this article.

**Conflict of Interest:** The authors declare that the research was conducted in the absence of any commercial or financial relationships that could be construed as a potential conflict of interest.

**Ethical Approval:** The data used in this study were publicly available. For being anonymized, this study does not require consent since it does not present risks for the research participants and was exempted from ethical approval by the Ethics Committee.

**Data and material availability:** The data used in this manuscript can be obtained from TabNet (https://datasus.saude.gov.br/informacoes-de-saude-tabnet/) and from the *Registro Brasileiro de Fibrose Cística* (Brazilian Cystic Fibrosis Registry) (http://portalgbefc.org.br/), or upon request to the authors.

**Authors’ Contribution:** LS: Conceptualization, Investigation, Methodology, Visualization, Writing – original draft, Writing – review & editing. MF: Conceptualization, Data curation, Investigation, Validation, Visualization, Writing – original draft, Writing – review & editing. FM: Conceptualization, Investigation, Methodology, Project administration, Resources, Supervision, Validation, Visualization, Writing – original draft, Writing – review & editing.

**Acknowledgments:** The authors are thankful to Silvana Aparecida Carvalho do Prado (MA in Language, Identity and Subjectivity, State University of Ponta Grossa), translator, for the translation of this article from Portuguese into English.

**Keywords:** Cystic Fibrosis; Epidemiology; Interstitial Pulmonary Diseases; Idiopathic Pulmonary Fibrosis; Medical Semantics

**Supplementary Tables**

| **Supplementary Table 1.** Distribution of deaths of individuals with cystic fibrosis [ICD (International Classification of Diseases): E84] in Brazil per year and age group (1996-2022) according to the Death Information System. | | | | | | | | | | | | | | |
| --- | --- | --- | --- | --- | --- | --- | --- | --- | --- | --- | --- | --- | --- | --- |
| **Year** | **Years of age** | | | | | | | | | | | | | |
|  | <1 | 1 to 4 | 5 to 9 | 10 to 14 | 15 to 19 | 20 to 29 | 30 to 39 | 40 to 49 | 50 to 59 | 60 to 69 | 70 to 79 | +80 | Unknown age | Total |
| **1996** | 17 | 10 | 7 | 3 | 9 | 4 | 2 | 2 | - | - | 1 | 1 | 2 | 58 |
| **1997** | 24 | 7 | 8 | 7 | 8 | 7 | 1 | 3 | 1 | 1 | 3 | 2 | – | 72 |
| **1998** | 16 | 10 | 14 | 3 | 4 | 2 | 2 | 2 | - | 1 | 2 | 1 | – | 57 |
| **1999** | 21 | 9 | 4 | 9 | 5 | 5 | 2 | 1 | 2 | 1 | 1 | 4 | – | 64 |
| **2000** | 22 | 8 | 12 | 9 | 1 | 7 | 2 | 2 | 1 | 3 | 7 | 3 | – | 77 |
| **2001** | 16 | 11 | 5 | 7 | 3 | 5 | 2 | 1 | 3 | 6 | 7 | 4 | – | 70 |
| **2002** | 22 | 9 | 10 | 11 | 8 | 9 | 3 | 1 | 1 | 1 | 1 | 1 | – | 77 |
| **2003** | 18 | 9 | 4 | 4 | 2 | 6 | 1 | 5 | 3 | 2 | 3 | 4 | – | 61 |
| **2004** | 16 | 9 | 5 | 10 | 7 | 12 | 2 | 4 | 2 | 2 | 3 | 3 | – | 75 |
| **2005** | 30 | 5 | 11 | 9 | 9 | 13 | 1 | 2 | 4 | 7 | 5 | 7 | – | 103 |
| **2006** | 21 | 4 | 9 | 12 | 9 | 11 | 4 | 8 | 10 | 8 | 10 | 5 | – | 111 |
| **2007** | 25 | 1 | 8 | 9 | 5 | 10 | 4 | 2 | 7 | 8 | 9 | 8 | – | 96 |
| **2008** | 20 | 2 | 7 | 12 | 6 | 9 | 3 | 4 | 4 | 4 | 15 | 7 | – | 93 |
| **2009** | 28 | 11 | 7 | 10 | 9 | 13 | 5 | 2 | 8 | 10 | 19 | 6 | – | 128 |
| **2010** | 15 | 4 | 13 | 7 | 8 | 10 | 11 | 6 | 4 | 14 | 19 | 14 | – | 125 |
| **2011** | 31 | 7 | 8 | 12 | 11 | 8 | 5 | 9 | 6 | 9 | 18 | 20 | – | 144 |
| **2012** | 22 | 4 | 6 | 14 | 6 | 18 | 4 | 9 | 9 | 10 | 16 | 12 | – | 130 |
| **2013** | 28 | 4 | 6 | 13 | 8 | 20 | 10 | 11 | 15 | 12 | 21 | 20 | – | 168 |
| **2014** | 17 | 7 | 5 | 20 | 21 | 20 | 12 | 5 | 18 | 17 | 23 | 26 | – | 191 |
| **2015** | 22 | 6 | 11 | 13 | 17 | 23 | 11 | 11 | 13 | 32 | 15 | 32 | – | 206 |
| **2016** | 17 | 10 | 12 | 21 | 12 | 22 | 14 | 14 | 17 | 19 | 37 | 30 | – | 225 |
| **2017** | 13 | 4 | 10 | 16 | 11 | 31 | 13 | 6 | 24 | 34 | 37 | 41 | – | 240 |
| **2018** | 24 | 7 | 4 | 19 | 16 | 20 | 7 | 11 | 15 | 27 | 37 | 44 | – | 231 |
| **2019** | 25 | 6 | 5 | 12 | 19 | 33 | 11 | 6 | 13 | 20 | 43 | 55 | – | 248 |
| **2020** | 17 | 5 | 10 | 15 | 11 | 19 | 7 | 12 | 18 | 30 | 48 | 32 | – | 224 |
| **2021** | 23 | 3 | 9 | 11 | 17 | 27 | 13 | 12 | 19 | 38 | 49 | 51 | – | 272 |
| **2022** | 20 | 8 | 15 | 13 | 10 | 25 | 15 | 8 | 31 | 36 | 59 | 51 | – | 291 |
| **Total** | 570 | 180 | 225 | 301 | 252 | 389 | 167 | 159 | 248 | 352 | 508 | 484 | 2 | 3837 |

Source: Death Information System (SIM, from Portuguese *Sistema de Informações sobre Mortalidade*, Open-Data-SUS). –, no cases recorded.

| **Supplementary Table 2.** Distribution of individuals with cystic fibrosis [ICD (International Classification of Diseases): E84] in Brazil into age groups considering their age at the time of the 2021 spirometry or anthropometry, N=3081. | |
| --- | --- |
| **Age group (years)** | **N (%)** |
| ≤05 | 862 (27.98%) |
| >05 and ≤10 | 702 (22.78%) |
| >10 and ≤15 | 554 (17.98%) |
| >15 and ≤20 | 382 (12.40%) |
| >20 and ≤25 | 223 (7.24%) |
| >25 and ≤30 | 112 (3.96%) |
| >30 and ≤35 | 94 (3.05%) |
| >35 and ≤40 | 47 (1.53%) |
| >40 and ≤45 | 35 (1.14%) |
| >45 and ≤50 | 26 (0.84%) |
| >50 | 34 (1.10%) |
| **Grouped age groups (years)** | **N (%)** |
| <18 | 2291 (74.35%) |
| ≥18 | 790 (25.64%) |

Source: Brazilian Cystic Fibrosis Registry (REBRAFC, from Portuguese *Registro Brasileiro de Fibrose Cística*), 2022.

| **Supplementary Table 3.** Distribution of deaths caused by ICD (International Classification of Diseases): J84.1 associated with interstitial pulmonary diseases including, mainly, idiopathic pulmonary fibrosis in Brazil per year and age group (1996-2022) according to the Brazilian Death Information System. | | | | | | | | | | | | | | |
| --- | --- | --- | --- | --- | --- | --- | --- | --- | --- | --- | --- | --- | --- | --- |
| **Year** | **Years of age** | | | | | | | | | | | | | |
|  | <1 | 1 to 4 | 5 to 9 | 10 to 14 | 15 to 19 | 20 to 29 | 30 to 39 | 40 to 49 | 50 to 59 | 60 to 69 | 70 to 79 | +80 | Unknown age | Total |
| **1996** | 88 | 13 | 5 | 5 | 9 | 32 | 61 | 84 | 101 | 199 | 229 | 194 | 1 | 1021 |
| **1997** | 77 | 21 | 6 | 5 | 9 | 29 | 45 | 84 | 108 | 208 | 233 | 152 | 2 | 979 |
| **1998** | 62 | 18 | 3 | 5 | 8 | 29 | 64 | 81 | 131 | 195 | 257 | 163 | 3 | 1019 |
| **1999** | 39 | 10 | 6 | - | 5 | 23 | 50 | 68 | 107 | 209 | 267 | 205 | 1 | 990 |
| **2000** | 42 | 9 | 6 | 1 | 10 | 22 | 35 | 53 | 101 | 183 | 290 | 247 | 2 | 1001 |
| **2001** | 36 | 8 | 4 | 5 | 6 | 24 | 47 | 77 | 119 | 215 | 323 | 254 | – | 1118 |
| **2002** | 34 | 12 | 4 | 5 | 6 | 14 | 37 | 59 | 152 | 249 | 385 | 279 | 2 | 1238 |
| **2003** | 13 | 6 | 3 | 3 | 6 | 18 | 46 | 73 | 143 | 252 | 407 | 374 | 2 | 1346 |
| **2004** | 22 | 6 | 2 | 1 | 2 | 12 | 38 | 93 | 148 | 269 | 423 | 414 | 1 | 1431 |
| **2005** | 12 | 15 | 2 | 3 | 4 | 17 | 46 | 82 | 147 | 273 | 496 | 447 | 1 | 1545 |
| **2006** | 17 | 9 | 3 | 8 | 6 | 24 | 37 | 84 | 198 | 319 | 542 | 529 | 1 | 1777 |
| **2007** | 19 | 6 | 8 | 8 | 6 | 23 | 63 | 86 | 205 | 371 | 606 | 560 | 2 | 1963 |
| **2008** | 26 | 7 | 2 | 6 | 7 | 24 | 42 | 101 | 202 | 411 | 631 | 610 | 5 | 2074 |
| **2009** | 15 | 12 | 7 | 6 | 11 | 35 | 54 | 112 | 227 | 391 | 647 | 623 | 2 | 2142 |
| **2010** | 21 | 12 | 8 | 5 | 7 | 24 | 37 | 103 | 215 | 422 | 665 | 789 | 2 | 2310 |
| **2011** | 16 | 6 | 1 | 7 | 9 | 24 | 39 | 95 | 226 | 442 | 711 | 804 | 2 | 2382 |
| **2012** | 20 | 9 | 4 | 4 | 5 | 23 | 58 | 116 | 212 | 441 | 732 | 832 | 2 | 2458 |
| **2013** | 24 | 5 | 4 | - | 10 | 20 | 45 | 99 | 266 | 508 | 719 | 895 | – | 2595 |
| **2014** | 13 | 6 | 3 | 5 | 9 | 22 | 54 | 104 | 264 | 509 | 834 | 931 | – | 2754 |
| **2015** | 24 | 8 | 1 | 3 | 8 | 24 | 53 | 89 | 237 | 548 | 844 | 1084 | – | 2923 |
| **2016** | 13 | 5 | 2 | 3 | 6 | 30 | 53 | 95 | 252 | 544 | 829 | 980 | 2 | 2814 |
| **2017** | 14 | 4 | 2 | 1 | 8 | 22 | 38 | 87 | 250 | 599 | 925 | 1203 | – | 3153 |
| **2018** | 22 | 12 | 4 | 6 | 3 | 23 | 43 | 96 | 291 | 621 | 989 | 1194 | 2 | 3306 |
| **2019** | 25 | 13 | 3 | 5 | 5 | 25 | 70 | 105 | 306 | 642 | 1074 | 1305 | – | 3578 |
| **2020** | 5 | 6 | 3 | 3 | 6 | 20 | 48 | 92 | 244 | 559 | 790 | 1134 | – | 2910 |
| **2021** | 13 | 4 | 4 | 6 | 3 | 19 | 42 | 129 | 256 | 593 | 937 | 1165 | – | 3171 |
| **2022** | 13 | 7 | 3 | 1 | 6 | 20 | 50 | 100 | 251 | 694 | 1110 | 1485 | 2 | 3742 |
| **Total** | 725 | 249 | 103 | 110 | 180 | 622 | 1295 | 2447 | 5359 | 10,866 | 16,895 | 18,852 | 37 | 57,740 |

Source: Death Information System (SIM, from Portuguese *Sistema de Informações sobre Mortalidade*, Open-Data-SUS). –, no cases recorded.
